# Radioprotective agents to prevent cellular damage due to ionizing radiation

**DOI:** 10.1186/s12967-017-1338-x

**Published:** 2017-11-09

**Authors:** Tyler A. Smith, Daniel R. Kirkpatrick, Sean Smith, Trevor K. Smith, Tate Pearson, Aparna Kailasam, Kortney Z. Herrmann, Johanna Schubert, Devendra K. Agrawal

**Affiliations:** 1 Department of Radiology, University of Utah, 30 North 1900 East #1A071, Salt Lake City, UT 84132 USA; 2 Department of Clinical & Translational Science, Creighton University School of Medicine, CRISS II Room 510, 2500 California Plaza, Omaha, NE 68178 USA; 3 Western University of the Pacific School of Medicine, CA Campus, 309 E. Second St, Pomona, CA 91766 USA; 4 Brigham Young University, Provo, UT 84602 USA

**Keywords:** Antioxidant, Computed tomography, Ionizing radiation, Medical imaging, Radioprotectant, Mitigators

## Abstract

Medical imaging has become a central component of patient care to ensure early and accurate diagnosis. Unfortunately, many imaging modalities use ionizing radiation to generate images. Ionizing radiation even in low doses can cause direct DNA damage and generate reactive oxygen species and free radicals, leading to DNA, protein, and lipid membrane damage. This cell damage can lead to apoptosis, necrosis, teratogenesis, or carcinogenesis. As many as 2% of cancers (and an associated 15,000 deaths annually) can be linked to computed tomography exposure alone. Radioprotective agents have been investigated using various models including cells, animals, and recently humans. The data suggest that radioprotective agents working through a variety of mechanisms have the potential to decrease free radical damage produced by ionizing radiation. Radioprotective agents may be useful as an adjunct to medical imaging to reduced patient morbidity and mortality due to ionizing radiation exposure. Some radioprotective agents can be found in high quantities in antioxidant rich foods, suggesting that a specific diet recommendation could be beneficial in radioprotection.

## Background

Medical imaging has become a central component of modern medical diagnosis. Over the past 10 years, increased utilization of X-ray examinations and computed tomography (CT) has led to corresponding increases in patient exposure to ionizing radiation raising awareness of the public to its deleterious effects. Despite notable decreases in the radiation dose associated with individual scans, increased utilization of medical imaging is a major contributor to radiation exposure and radiation-associated pathology [1, 2]. Long-term studies of the Second World War atomic bomb survivors in Japan; i.e. those with significant radiation exposure, have been found to have an increased incidence of both leukemia and solid cancers [1]. Based on the linear no threshold model, imaging-related radiation, while certainly less dramatic than an atomic explosion, may pose significant radiation-related risks. The risks associated with radiation exposure are known to be more pronounced in younger patients. This fact is demonstrated by the increased prevalence of leukemia and solid tumors among the pediatric atomic bomb survivors compared to those who underwent the same radiation exposure at an older age [1]. Gilbert et al. [2] showed a dose-dependent relationship between radiation exposure and leukemia, breast cancer, thyroid cancer, and other solid tumors. Ionizing radiation has immediate, measurable deleterious effects on cells, including increasing reactive oxygen species (ROS), generation of single stranded DNA breaks (SSBs), and double stranded DNA breaks (DSBs) [1, 3].

Several authors have proposed using a variety of agents to modulate the cellular damage associated with radiation exposure. It is postulated, for example, that antioxidants or glutathione-elevating compounds may be able to reduce DNA damage, theoretically reducing carcinogenesis post-radiation [4, 5]. Although many studies have demonstrated potential benefits for a variety of agents, radioprotective compounds are not routinely administered to patients before or after medical imaging [6]. The aim of this review is to summarize and critically evaluate the recently published findings in the literature that investigated the use of radioprotective agents to avoid radiation-associated cell damage.

Ionizing radiation is widely used in medical diagnostics, cancer-related therapy, and has additional industrial applications [7]. Known hazards associated with human exposure to ionizing radiation include: induction of cellular death, genetic mutations, and carcinogenesis [7]. In addition to direct cellular effects, radiation exposure can also damage cells through the generation of reactive oxygen species (i.e. hydrogen peroxide, lipid hydroperoxides, superoxide, hydroxide, hydride, and peroxynitrite). Reactive oxygen species (ROS) are formed when ionizing radiation is absorbed by small molecules, primarily water, surrounding cellular bio-macromolecules. These ROS react with cellular contents, including DNA and proteins [7].

**Fig. 1:**
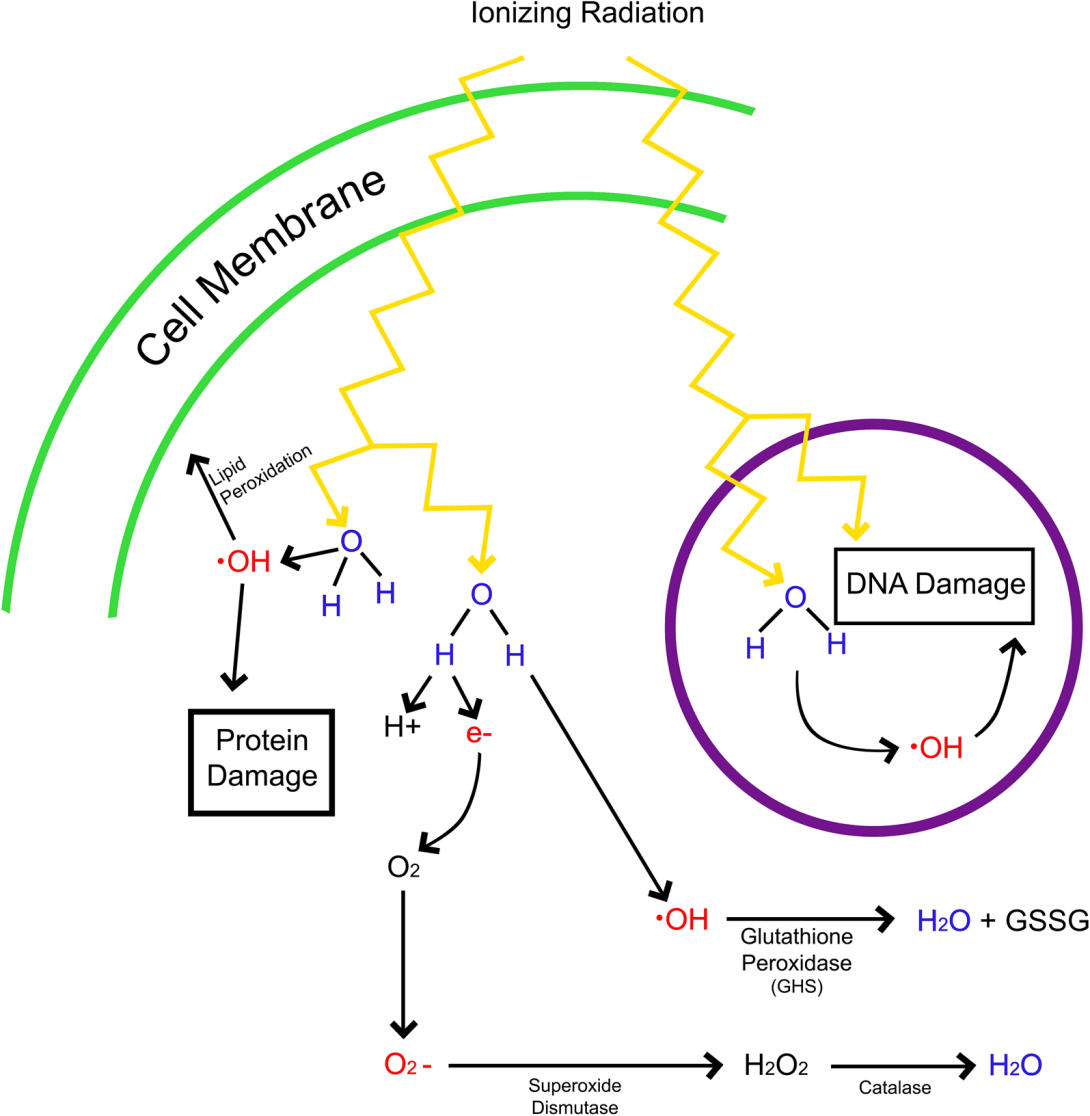
Generation of reactive oxygen species (ROS) in response to ionizing radiation. Ionizing radiation induces damage of cellular structures in two primary ways: direct damage to DNA and generation of free radical-containing reactive molecules. Free radicals are generated through the interactions between ionizing radiation and small oxygen containing molecules (including water). These interactions commonly form hydroxide and generate free electrons. Free electrons can then interact with intracellular oxygen to form superoxide. Free radicals that are generated by ionizing radiation can react with DNA, lipid membranes, and proteins causing damage and/or dysfunction to various cellular structures. The cell has mechanisms designed to mitigate and manage damage from free radicals. Hydroxide ions are reduced by the enzyme glutathione peroxidase and superoxide ions are reduced to hydrogen peroxide by superoxide dismutase. Hydrogen peroxide generated by superoxide dismutase is used by catalase to generate water. Significant damage to cellular structures occurs when ionizing radiation-induced generation of radicals out-paces the cell’s ability to clear these reactive molecules

**Fig. 2:**
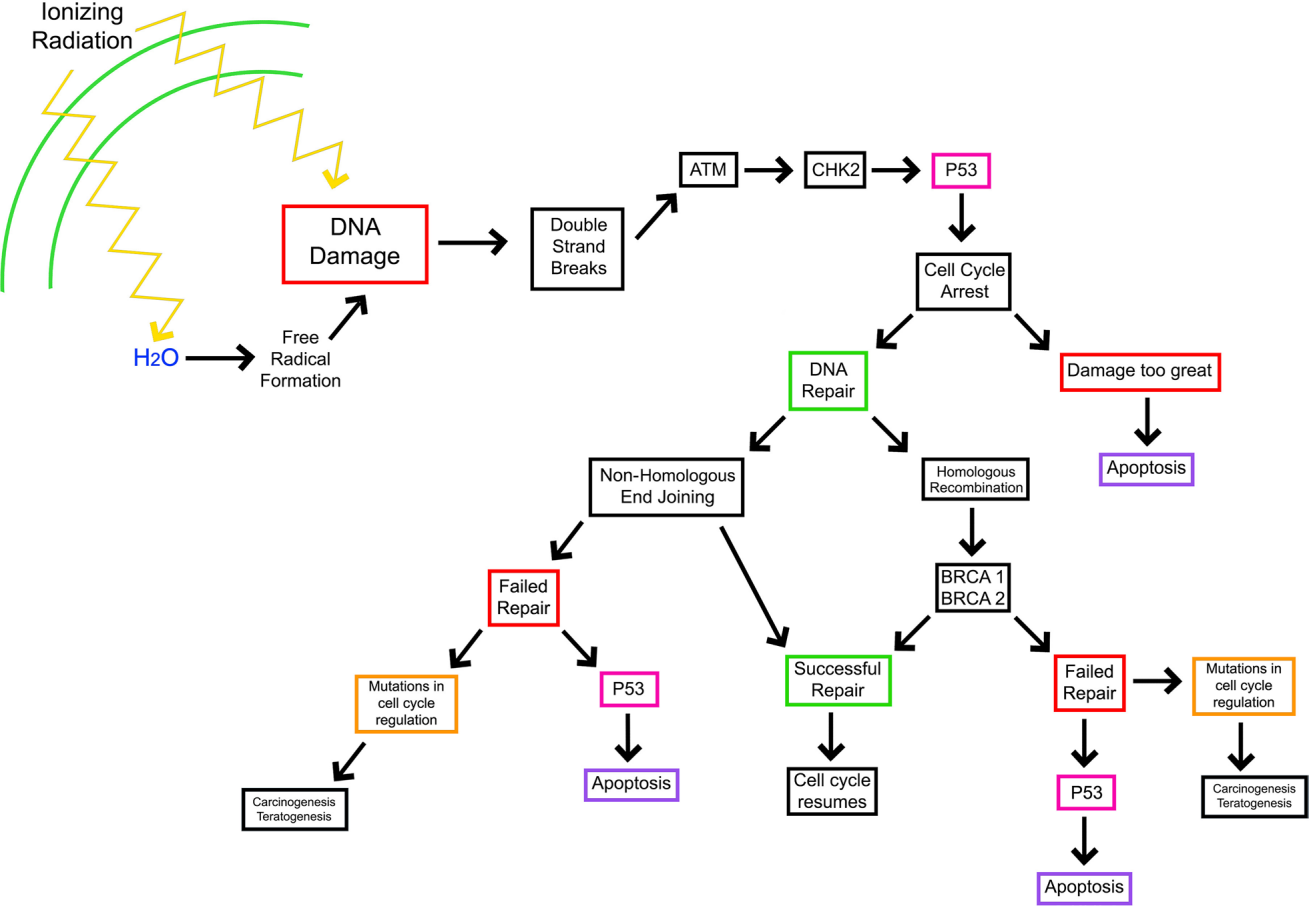
Downstream molecules and effects following DNA damage due to ionizing radiation. Ionizing radiation causes damage to DNA both directly and indirectly. Indirect damage occurs through the radiation-associated formation of free radicals. Double-stranded breaks (DSBs) are the most common form of DNA damage associated with ionizing radiation. After DSBs are generated, a cascade of enzymatic processes is triggered to allow for DNA repair or to induce apoptosis. This process includes the activation of p53 and the induction of cell cycle arrest. If the damage exceeds the cell’s ability to repair itself, either apoptosis or necrosis will occur. Alternatively, there are two common mechanisms of DSB repair: Non-homologous end joining and homologous recombination. In homologous recombination, the enzymes BRCA 1 and BRCA 2 are activated and initiate repair. If repair is successful, the cell cycle can resume. If homologous recombination is unsuccessful the cell will likely undergo apoptosis. Importantly, failure of these processes in the setting of significant mutations in cell cycle regulation or the apoptotic pathway can lead to carcinogenic transformation. In non-homologous end joining, as the name suggests, non-homologous ends are joined together to mitigate DNA damage. This can lead to significant mutations in cell cycle regulation and result in carcinogenic transformation

The cell responds to increased concentrations of free radicals by generating natural antioxidants (including superoxide dismutase, glutathione, catalase) which can minimize or eliminate free-radical induced damage to cellular structures (Fig. 1). Glutathione peroxidase primarily catalyzes the conversion of hydroxide ions to water. Superoxide dismutase converts superoxide ions to hydrogen peroxide, which is then converted to oxygen and water by catalase. Superoxide dismutase exists in several different isoforms, each of which is specialized to specific areas of the cell [8]. When exposed to increasing levels of ionizing radiation, the cell increases expression of antioxidant enzymes [8]. When, however, the level of ROS overwhelms these cellular defenses, the cell will sustain damage (in a dose-dependent manner) that can lead to carcinogenesis, teratogenesis, necrosis, or apoptosis (Fig. 2).

Administering radioprotective agents has been proposed as one way to decrease radiation-related deleterious effects on cells. Antioxidants have the potential to act as free radical scavengers, and thus reduce some DNA damage caused by ionizing radiation [4, 7, 9, 10]. Theoretically, this intervention would allow cellular defenses to keep pace with the free radicals generated by radiation exposure (assuming the intracellular level of antioxidants is sufficient at the time of radiation exposure). Radioprotective compounds may suppress free radical formation, remove free radicals, induce natural radioprotector production (such as superoxide dismutase, glutathione peroxidase, and catalase), enhance DNA repair, reduce the post-radiation inflammatory response, or even delay cellular division allowing more time for cells to repair or undergo apoptosis [10] (Table 1). Although radioprotective substances have been shown to be effective at decreasing the side effects of radiation therapy, there are currently no radioprotectants used in diagnostic radiology.

**Table 1:** Potential underlying mechanisms/effects of the radioprotective agents that decrease DNA damage

Decreased DNA damage	Induction of natural antioxidants (glutathione, superoxide dismutase, catalase)	Free radical scavenging activity	Decreased lipid peroxidation	Improved survival in mice and rat models	Inhibit apoptosis	Direct cell cycle effects	Reduction in post radiation exposure inflammatory response
*N*-acetyl cysteine, vitamin C, R-α-lipoic acid, l-selenomethionine, l-ascorbic acid (vitamin C), 6-*o*-palmitoylascorbate, carnosic acid, green tea extract, apigenin, diosmine, rosmarinic acid, δ-tocopherol, rutin, amifostine, carvacrol, inapoyl-E-glucoside, quercetin-3-*O*-rhamnoside-7-*O*-glucoside, quercetin-3-*O*-rhamnoside, luteolin-7-*O*-(2-apiosyl)-glucoside, quinic acid, chlorogenic acid, curcumin and analogs, dendrodoine analog, resveratrol, cinnamic acid, epigallocatechin-3-gallate GANRA-5, sodium ascorbate, α-lipoic acid, coenzyme Q10, vitamin E, isofraxidin, melatonin, watermelon juice, lactoferrin, guanine nucleotides, black grape juice, caffeic acid phenethyl ester, kukoamine A, α-tocopherol acetate, 17-β-estradiol, β carotene, eukarion, acteoside	*N*-acetyl cysteine, curcumin, resveratrol, melatonin, watermelon juice, guanine nucleotides, black grape juice, cafeic acid phethyl ester, kukoamine A	Carvacrol, inapoyl-E-glucoside, quercetin-3-*O*-rhamnoside-7-*O*-glucoside, quercetin-3-*O*-rhamnoside, luteolin-7-*O*-(2-apiosyl)-glucoside, GANRA-5, Sodium ascorbate, α-lipoic acid, coenzyme Q10, vitamin E, melatonin, guanine nucleotides, 17-β-estradiol	Vitamin C, inapoyl-E-glucoside, quercetin-3-*O*-rhamnoside-7-*O*-glucoside, quercetin-3-*O*-rhamnoside, luteolin-7-*O*-(2-apiosyl)-glucoside, carvacrol, curcumin, dendrodine analog, epigallocatechin-3-gallate, black grape juice, melatonin, watermelon juice	Watermelon juice, black grape juice, *N*-acetyl cysteine, guanine nucleotides, GANRA-5, lactoferrin amifostine	Carvacrol, acteoside, isofraxidin, caffeic acid phenethyl ester, kukoamine A, vitamin E, atorvastatin, amifostine	Resveratrol, melatonin	Carvacrol, GANRA-5, melatonin, Caffeic acid phenethyl ester

Most agents presented in this paper were found to decrease DNA damage, although a mechanism was not always proposed. Free radical scavenging and induction of natural antioxidants were the most common proposed mechanisms. A decrease in lipid peroxidation was also common. A reduction in free radicals via direct scavenging activity or induction of natural antioxidants would likely lead to both decreased DNA damage and decrease in lipid peroxidation. Only a few of the agents included in this review were shown to have direct cell cycle effects; however, this was not a common area of investigation in the literature. Notably, studies on some agents also showed a reduction in the post radiation exposure inflammatory response. Some authors have suggested that this inflammatory response could be due to depletion of natural antioxidants, leading to cell injury, cell death, and associated inflammation. Thus, it is likely that agents which act as free radical scavengers or induce natural antioxidants would also lead to a reduction in post radiation inflammatory response

To summarize existing candidates for clinical radioprotectors, we conducted a literature review using a Pubmed/MEDLINE search with key phrases including: “antioxidants in medical imaging”, “antioxidants in radiotherapy”, “antioxidant radiation”, “radioprotective agents”, “radioprotective radiotherapy”, “radioprotective medical imaging”, and “radioprotection.” To be included, the articles were required to be peer-reviewed primary research articles published in the past 10 years that investigated one or more substances as potential radioprotective agents. This article represents a summary and critical analysis of the selected articles investigating radioprotection. Moreover, this article summarizes key findings relevant to the following clinical question: can radioprotectants be used in diagnostic imaging to reduce DNA damage?

### Findings from in vitro studies

#### In vitro: human lymphocytes

The preponderance of the literature on radioprotective agents comes from studying human lymphocytes in vitro before and after exposure to radiation. Occasionally, this includes taking blood samples from patients after they undergo clinical imaging. Usually, these studies quantify radiation-induced double-stranded DNA breaks (DSBs) via γ-H2AX foci (γH2AX). For example, Brand et al. [9] showed that several antioxidants, if administered before exposing human blood to radiation, could lower the incidence of DSBs in lymphocytes. Importantly, *N*-acetyl cysteine (NAC) and vitamin C lowered DSBs by 43 and 25% respectively, which was significantly more than any of the other agents studied [9].

Interestingly, despite individual agents showing promise as radioprotectants, none of the combinations tested by the authors showed an additive effect when multiple agents were used together [9]. This study supports using antioxidants, particularly NAC and vitamin C analogs, to prevent radiation-associated DNA damage. Like Brand et al. [9], Kuefner et al. [11] conducted a study investigating the effects of a mix of antioxidants (calcium ascorbate, d-alpha tocopheryl succinate, carotenoids, NAC, R-α-lipoic acid, l-selenomethionine) on in vitro human lymphocytes. Kuefner et al. [11] did this in two ways: first, in vitro lymphocytes were treated with antioxidants, then irradiated. Second, blood samples were obtained 15, 30, 60 min, 2, 3, 5 h after ingestion of a pill containing the study antioxidants, then the lymphocytes were irradiated. While administering antioxidants after irradiation did not lead to a reduction in DSBs, pretreatment with antioxidants caused significant reductions in DSBs, with a 23% reduction after 15 min and a 58% reduction if administered 60 min before irradiation [11]. This study had clinical value because the experimental radiation exposure was comparable to that received during a CT scan. In another study, NAC and vitamin C were both administered immediately before and after patients were exposed to X-ray. Patients’ blood was then drawn and DSBs were measured in lymphocytes. Both vitamin C and NAC were found to decrease DSBs, as measured by γH2AX, compared to controls [12]. In each of these studies, NAC significantly decreased radiation-related DNA damage in human lymphocytes.

An important study by Reliene et al. [13] looked at the effects of NAC in human, murine, and yeast models. While this study did find that NAC reduced γH2AX foci (a surrogate for DSBs), it also noted that cell colony survival was unchanged in yeast and human lymphocytes [13]. In other words, while NAC decreases DNA damage, it does not necessarily prevent apoptosis or necrosis. This finding may have important potential implications: NAC may decrease the incidence of DNA damage without interfering with the purposeful death of cancerous or pre-cancerous cells [13]. NACs ability to decrease or avoid DNA damage without protecting cells from apoptosis may increase its clinical value (relative to other antioxidants).

Other studies have focused specifically on vitamin C and its derivatives. In a 2014 study, Xiao et al. [3] exposed human lymphocytes to radiation after being plated for 3 h with differing concentrations of one of two vitamin C derivatives: 6-*O*-palmitoylascorbate (PlmtVC) or l-ascorbic acid (l-AA). As a radioprotective agent, PlmtVC outperformed l-AA, showing that not all vitamin C derivatives are equally efficacious as antioxidants [3]. PlmtVC significantly decreased lipid peroxidation and protein carbonylation compared to controls while also elevating endogenous glutathione [3]. PlmtVC also significantly reduced the total number of DSBs compared with either controls or l-AA [3]. While some studies have shown vitamin C to have radio-protective activity, other studies have shown vitamin C to potentiate radiation-induced damage [14–16]. This dichotomy makes vitamin C a controversial agent for clinical use as a radioprotectant.

Although vitamin C and NAC have shown promising results, a multitude of other agents have been studied using human lymphocytes in vitro. Alcaraz et al. [17] conducted a study to assess 10 different antioxidant compounds (carnosic acid, green tea extract, apigenin, diosmine, rosmarinic acid, l-ascorbic acid, δ-tocopherol, rutin, amifostine, dimethylsulphoxide) as candidates for radioprotectants against chromosomal damage caused by ionizing radiation (with DSMO as the control and vehicle). When compared to irradiated controls, all compounds showed a decrease in DNA damage, with the greatest effects seen in rosmarinic acid, carnosic acid, δ-tocopherol (vitamin E), and apigenin [17]. Less effective agents included l-ascorbic acid, amifostine, green tea extract, rutin, and diosmine [17]. This same pattern was also seen in terms of magnitude of radioprotection provided by these agents [17].

Arivalagan et al. [18] investigated carvacrol (CVC) as a potential radioprotective agent due to its safety for consumption (it is a common food additive), anti-inflammatory, and antioxidant properties. In this study, lymphocytes were collected from healthy individuals and then treated with DMSO or CVC prior to radiation. Not surprisingly, as radiation dose increased cell survival decreased and DNA damage increased in the control groups [18]. Lymphocytes pretreated with CVC experienced a statistically significant rise in the lethal dose of radiation they could tolerate compared to controls. CVC-treated lymphocytes also showed a significant decrease in DNA damage as well as decreased lipid peroxidation and apoptosis [18]. CVC appears to decrease free radical damage in two ways: as an antioxidant and as a free radical scavenger [18]. CVC holds promise as a radioprotective agent with few side effects or toxicity.

Phenolic glycosides, which occur naturally in plants, have also been shown to have antioxidant properties [19]. Materska et al. [19] investigated several phenolic glycosides: sinapoyl-E-glucoside (sEg), quercetin-3-*O*-rhamnoside-7-*O*-glucoside (q3Or7Og), quercetin-3-*O*-rhamnoside (q3Or) and luteolin-7-*O*-(2-apiosyl)-glucoside (l7O2ag). The authors used human lymphocytes obtained from healthy human donors, and then exposed them to one of the phenolic glycosides before irradiation with X-rays. Researchers found that q3Or showed the highest radioprotective effect, with a 50% reduction in DNA damage compared to controls. Importantly, in this study these substances did not show any toxic effects against human lymphocytes [19]. The phenolic glycosides were also noted to have excellent antiradical activities [19]. In this study, compounds with greater superoxide radical scavenging capabilities also demonstrated better radioprotective effects [19]. The radioprotective effects of other phenolic glycosides including quinic and chlorogenic acid have also been studied on human lymphocytes in vitro. In one study, lymphocytes were exposed to differing doses of X-ray radiation and treated with differing concentrations of either quinic acid, chlorogenic acid, or a sham control. This study found that lymphocytes pretreated with both quinic acid and chlorogenic acid prior to irradiation had significant decreases in DNA damage as measured by the genetic damage index [20]. In the case of chlorogenic acid, however, there was no significant changes in the genetic damage index in the lower X-ray radiation dose range [20]. Quinic acid also decreased the percentage of cells damaged by radiation [20]. Quantitatively, the magnitude of protection (based on the genetic damage index) was calculated to be 5.99–53.57% for quinic acid and 4.49–48.15% for chlorogenic acid [20]. The radioprotective efficacy of quinic acid and chlorogenic acid seems to be comparable to other phenolic phytochemicals like curcumin, caffeic acid, hesperidin, vanilla, and resveratrol [20]. The observed effects of both quinic acid and chlorogenic acid may be related to vicinal hydroxyl groups on an aromatic residue which may possess an anti-mutagenic, anti-carcinogenic and antioxidant effects in vitro [20].

Cinnamic acid is a phenolic substance obtained from cinnamon oil, and has been shown to have antioxidant properties. Cinkilic et al. [21] investigated the radioprotective effects of cinnamic acid against X-ray-induced genomic instability in human lymphocytes. They found that cinnamic acid-treated lymphocytes had a significant decrease in DNA DSBs (range from 16 to 55% reduction) compared to controls [21]. Pretreatment with cinnamic acid also reduced total genetic damage [21]. Cinnamic acid alone did not increase DSBs or other DNA damage, suggesting it is not genotoxic [21]. The authors found that cinnamic acid decreased DNA damage induced by irradiation with X-rays by reducing the intracellular ROS level through its free-radical scavenging properties [21]. As a group, phenolic glycosides include many agents that show potential for decreasing radiation associated DNA damage.

In a recent study, Soltani et al. [22] investigated the use of free curcumin and a novel dendrosomal nanoformulation of curcumin (DNC) in human leukemia cells. Prior studies have indicated that high concentrations of curcumin may induce apoptosis in human leukemia cell lines via activation of JNK/ERK/AP1 pathways. Interestingly, curcumin is believed to be an antioxidant at lower concentrations and a pro-oxidant at higher concentrations [22]. The authors of this study found that pretreatment of lymphocytes with low concentrations of free curcumin had a protective effect on irradiated cells via enhanced antioxidant effects. However, low concentrations of DNC lead to decreased cell viability and survival [22]. The authors concluded that low concentrations of free curcumin protected cells from radiation via increased scavenging of free radicals, activation of Nrf2 pathway (thus leading to increased expression of total antioxidant and thiol levels) and upregulation antioxidant gene expression [22]. Meanwhile, DNC induced apoptosis [22]. Another study looking at curcumin on irradiated lymphocytes, Srinivasan et al. [23] also found that there was a significant decrease in lipid peroxidation in all groups pretreated with curcumin, and significant increases in reduced glutathione. Both effects were dose-dependent: they were most pronounced in the highest concentration pretreatment groups [23]. Pretreatment with curcumin also lead to a significant increase in the activities of superoxide dismutase, catalase, and glutathione peroxidase after gamma-irradiation [23]. Not only did curcumin show significant anti-oxidative and anti-lipid peroxidative properties, but pretreated groups were found to have less overall DNA damage [23]. Given the reduced lipid peroxidation, improved antioxidant status, and reduced DNA damage in curcumin pretreated groups, the authors concluded that curcumin may induce the transcriptional factors for oxidative stress-related gene expression [23]. These studies support using certain curcumin analogs for mitigating the deleterious effects of radiation.

Although curcumin is widely accepted as a radioprotectant, its clinical application is hampered due to its limited bioavailability. Nguyen et al. [24] used curcumin-encapsulated liposomes to deliver curcumin to ^60^cobalt gamma radiation-damaged human lymphocytes and found that the curcumin-encapsulated liposomes had a dose-dependent radioprotective effect, with higher doses of curcumin being more radioprotective up to 30 μg/mL [24]. These investigators concluded that encapsulation with liposomes could increase the bioavailability of curcumin adding to its clinical use and may be effective as a delivery system for other radioprotective phytochemicals.

Kalpana et al. [25] investigated the use of a dendrodoine analog (DA), derived from marine alkaloids extracted from the tunicate *Dendrodoa grossularia*. DA has been reported to be cytotoxic to lymphoma cells in culture, and it also contains aminothiazole compounds which have anti-tumor and antioxidant properties. The investigators incubated lymphocytes with differing concentrations of DA and then exposed them to X-rays. Compared to the control groups DA treated groups had less DNA damage and lipid peroxidation [25]. The authors concluded that this action was likely through the antioxidant effects of DA; however, the exact mechanisms by which DA acts are still unknown [25]. Since this study showed that human lymphocytes cultured in the presence of DA suffered less radiation-induced damage, DA is a potential candidate for pretreatment before ionizing radiation exposure.

Many researchers have focused on compounds found in plants and phytochemicals (in addition to curcumin and others discussed) as potential radioprotectants against ionizing radiation. In one study, Davari et al. [26] collected blood from volunteers who drank a green tea for five consecutive days prior to blood draws. The whole blood sample was then exposed to gamma radiation. It was found that the lymphocytes collected 3 h after drinking green tea showed a significant decrease in DNA damage compared to controls [26]. Prasad et al. [27] explored the effects of ferulic acid on cultured lymphocytes. These investigators exposed lymphocytes pretreated with varying concentrations of ferulic acid to gamma radiation, and found that the treatment with ferulic acid for 30 min prior to radiation exposure resulted in a significant reduction in DNA damage compared to non-treated controls and that higher concentrations provided more protective effects [27]. Ferulic acid was proposed to work by preventing radiation-induced decrease in the activity of superoxide dismutase, catalase, and glutathione reductase [27]. Rodeiro et al. [28] conducted a study on the extract of *Mangifera indica* L. (mango) to evaluate its potential radioprotective effects in human lymphocytes. The lymphocytes were incubated with varying concentrations of *Mangifera indica* L. *extract* followed by exposure to gamma rays. Incubation of lymphocytes with *Mangifera indica* L. *extract* 1 h before exposure to gamma radiation reduced DNA damage [28]. This list is not all-inclusive, but rather suggests that plants and phytochemicals could be a rich source of potential radioprotectants.

A wide array of agents has been shown to decrease radiation-induced DNA damage in human lymphocytes. While many of these agents may ultimately have clinical value, it has not been definitively established that decreasing DNA damage in WBCs leads to clinically significant benefits. Future research, particularly long-term trials, will be necessary to demonstrate any concrete clinical benefit related to radioprotective agents.

#### In vitro: human non-lymphocyte cells

Although most studies investigating radioprotective effects have been conducted on human lymphocytes in vitro, many studies have also been done on other non-lymphocyte human cell types. For example, Monzen et al. [29] performed a study in which they isolated CD34+ cells and separated them into granulocyte and erythroid precursors. The cells were then pretreated with epigallocatechin-3-gallate (EGCg) prior to irradiation. EGCg is a natural antioxidant found in most teas. The addition of EGCg before irradiation significantly improved the survival of erythroid progenitors at low radiation doses; however, the same effects were not observed in granulocyte precursors [29]. These findings suggest that a low concentration of EGCg provides more protection from radiation damage in erythropoiesis than granulopoiesis. The authors reported that EGCg works as an antioxidant by trapping free radicals thus preventing lipid peroxidation and DNA damage [29]. These finding suggest that EGCg may work particularly well with hematopoietic recovery after irradiation, and may be a more cost-effective treatment than currently available medications [29]. Prior studies have shown that after drinking 1–2 cups of tea, the mean peak plasma EGCg level was like the concentrations used for their study, supporting simple dietary modifications as a means of radioprotection [29]. This observation is broadly applicable to other antioxidants: they are readily available in healthy diets, an observation that underscores the value of good nutrition to those undergoing radiation exposure.

Resveratrol is a known antioxidant and free radical scavenger, and is also known to have significant cell cycle effects, including stabilization of p53 and alterations of pro- and anti-apoptotic protein concentrations [30, 31]. One study found resveratrol to have a pro-apoptotic effect on leukemia, mammary, and epidermoid cell lines, and growth-inhibitory activity in some human cancer cell lines [32]. Firouzi et al. [30] found that when resveratrol was administered before radiation, DNA damage and colony death was increased in resveratrol-treated glioblastoma cells relative to controls. Firouzi et al. [30] further showed that resveratrol binds to HIF1-α in hypoxic conditions (often found in neoplastic growth), leading to stabilization of p53 and decreased function of the vasculogenic VEGF [30]. In other words, resveratrol decreased new vascular growth to the glioblastoma cells while simultaneously stabilizing intra-cellular mechanisms for detecting and killing genetically mutated cells. The mechanisms for increased cell death described by Firouzi et al. [30] are complemented by effects described by Carsten et al. [31] who found that resveratrol decreased expression of anti-apoptotic proteins like BCL2 and increased expression of pro-apoptotic proteins like BAX in cancer cells. Resveratrol, then, may ultimately prove to have value in cell-cycle or ROS-related disease, including radiation-induced cellular damage.

As skin is usually the first tissue that encounters ionizing radiation, the role of human fibroblasts have also been studied in the context of radioprotection. In a recent study, Bao et al. [33] used human fibroblasts to investigate the role of hemin in the radioadaptive response. In fibroblasts, the activity of heme oxygenase 1 (HO1) was observed to increase with exposure to radiation. When a competitive inhibitor of HO1 was given, this radioadaptive response was observed to decrease [33]. Conversely, when cells were treated with hemin, an inducer of HO1, radiation-related DNA damage decreased by nearly 50% [33]. These results suggest that upregulation of HO1 could improve cell viability after radiation exposure, making hemin a potential candidate for radioprotection [33]. Another substance studied using human fibroblasts is acteoside, a known antioxidant and anti-inflammatory. Acteoside is a phenylethanoid glycoside derived from the *Cistanche salsa* plant of northwest China. Yang et al. [34] studied the effects of acteoside on irradiated human fibroblasts. Pre-incubation with acteoside decreased the generation of ROS and led to a significant decrease in apoptosis compared to controls. Acteoside was also observed to down-regulate pro-caspase 3, decrease expression of Bax, and increase expression of BCl2 compared to controls [30]. Perhaps most significantly, Yang et al. [34] showed that acteoside leads to a significant increase in the phosphorylation of ERK and JNK, suggesting it could play a role in cell cycle regulation and increasing its potential as a radioprotective agent.

Pei et al. [10] studied the oxazolone derivative GANRA-5 (a known free radical scavenger) on human lung fibroblasts. Interestingly, in this study GANRA-5 was shown to be radioprotective in a variety of radiation settings (X-ray, carbon ion beams, microwave, UV light). The authors also noted that fibroblasts protected with GANRA-5 had significantly lower formation of gamma-H2AX foci compared to controls after exposure to X-ray radiation [10]. The potential combination of tolerability and efficacy make GANRA-5 an important radioprotective agent for future studies and, perhaps, clinical use.

Wan et al. [35] used antioxidants as radioprotective agents against radiation-induced oxidative stress in human epithelial cells. The antioxidants they studied included NAC, ascorbic acid, sodium ascorbate, alpha-lipoic acid, coenzyme Q10, l-selenomethionine, and vitamin E. In this experiment, Wan et al. [35] exposed human breast epithelial cells to X-ray and gamma-ray radiation. Before radiation exposure, cells were treated with a medium containing a single antioxidant, a combination of antioxidants, or an antioxidant-free control. They found that while individual antioxidants provided varying degrees of protection against X-rays and gamma-ray induced DNA damage, combinations of several antioxidants produced the most profound reduction in DNA damage (94.7% reduction against X-ray radiation and 100% reduction against gamma-ray radiation) [35]. Wan et al. [35] also noted that water soluble free radical scavengers (such as NAC, ascorbic acid, sodium ascorbate, and α-lipoic acid) were the most effective at reducing DNA damage. They hypothesize that this is because many free radicals are likely generated in an aqueous environment, and water soluble antioxidants will be present at the source of free radical generation [35]. In contrast to some prior studies [9], Wan et al. [35] emphasized that the combination of antioxidants was more effective than individual antioxidants in protecting against radiation-induced oxidative stress. They expected this result, and suggested that antioxidants can replenish one another and increase the total pool of antioxidants available to react with free radicals [35]. Further studies should be pursued to establish whether additive or synergistic interactions occur among radioprotective antioxidants.

**Fig. 3:**
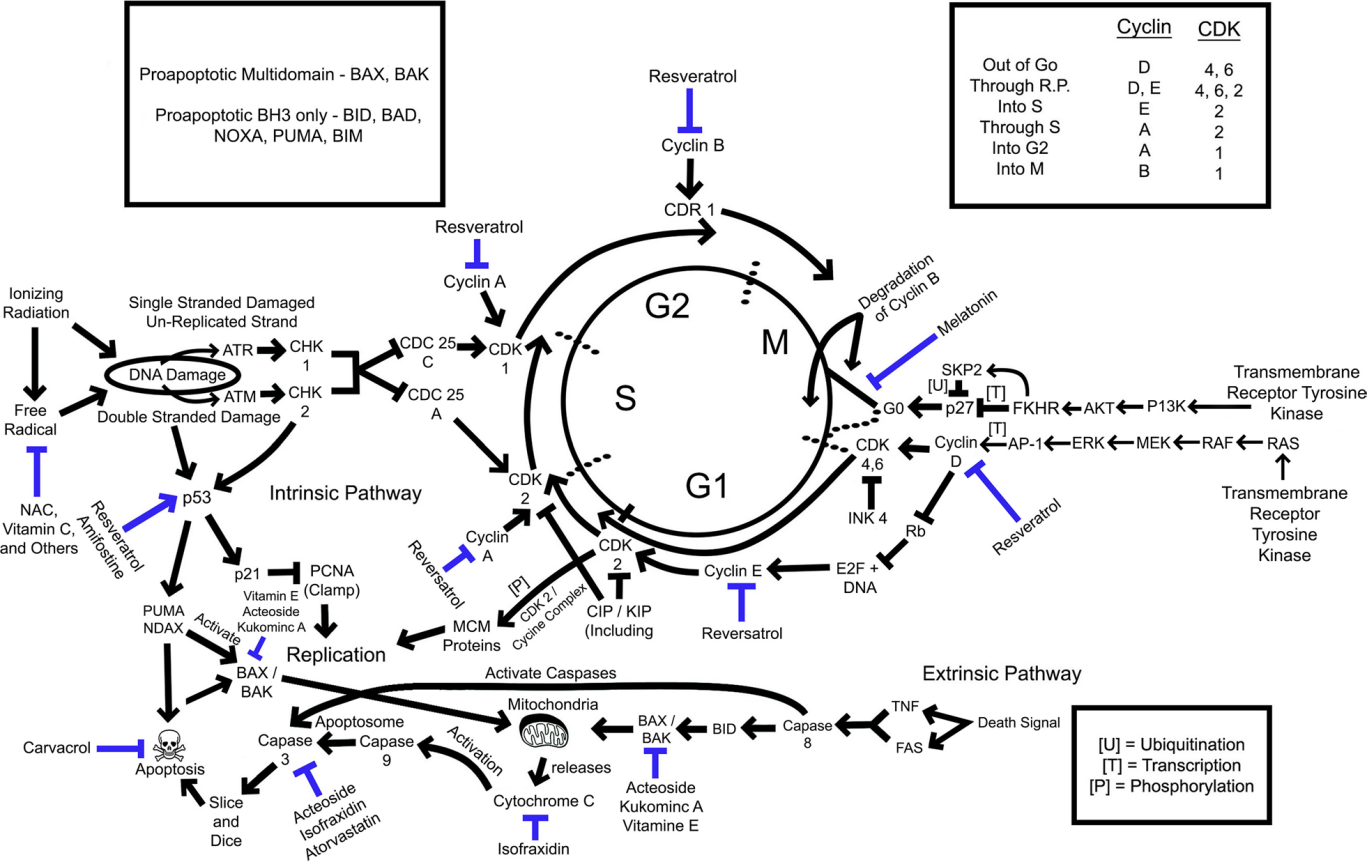
Proposed effects of radioprotectant agents in the cell cycle. This figure presents an overview of the cell cycle and includes the proposed effects of the radioprotectant agents discussed in this review. Resveratrol was one of the most widely studied in this area, having effects on cyclin expression and thus cell cycle progression. It also was shown to induce p53 [30–32, 37]. Additionally, amifostine was shown in one study to induce expression of p53 and inhibit its degradation. Melatonin was shown to inhibit progression to the G0 phase in endothelial cells. Carvacrol had an inhibitory effect on cellular apoptosis. Vitamin E, kukoamine, and acteoside inhibited pro-apoptotic proteins Bax and Bak. Acteoside shown to inhibit expression of caspase 3, and thus decrease apoptosis. Similarly, atorvastatin was shown to decrease expression of caspase 3. Isofraxidin both inhibited cytochrome C and caspases, specifically caspase 3, leading to a reduction in apoptosis. Most authors proposed that the studied radioprotectants may act as free radical scavengers or inducers of natural antioxidants (see Table 1)

Li et al. [36] conducted an experiment looking at isofraxidin (IF), an ROS scavenger, to better elucidate the process of ROS-induced apoptosis. In this experiment, they exposed U937 lymphoma cells pretreated with IF to high dose ionizing radiation. The result was a significant reduction in apoptosis (13.7% reduction) in IF-treated cells and better cell survival at 6 and 24 h [36]. Decreased apoptosis and improved cell survivability corresponded to a reduction in ROS generation compared to non-IF controls [36]. Li et al. [36] hypothesized that the reduction in apoptosis was related to a reduction in ROS. IF also prevented the activation of caspase-3, down-regulated the expression of Bax, and inhibited the release of cytochrome C from the mitochondria of irradiated cells (Fig. 3) [36]. Oxidative stress is also an activator of MAPKs (known activators of apoptosis) and activates a pathway including JNK and p38 via phosphorylation [36]. IF-treated cells inhibited the phosphorylation of JNK and p38, suggesting that IF might also play an anti-apoptotic role through MAPK p38/JNK pathway [36]. Li et al. [36] also looked at the effects of IF on the extrinsic apoptosis pathway by measuring FAS death receptor expression and caspase-8 activation. They found that IF-treated cells had a significant decrease in FAS externalization and caspase-8 activation [36]. Finally, the authors looked at two other leukemia cell lines (Molt-4 and HL60) to investigate their effect on p53 expression. They found no differences in p53 expression between irradiated cells treated with IF and controls, suggesting that IF likely acts through a p53-independent mechanism. They concluded that IF plays an anti-apoptotic role in response to ionizing radiation by decreasing hydroxyl radical formation, Bax-mitochondrial pathway, and JNK/p38 MAPK activation [36]. More work should be done to determine if the molecular mechanisms whereby IF mediates its anti-apoptotic effect are applicable to other radio-protective agents. Moreover, this study underscores several potential molecular targets for radio-protective interventions.

#### In vitro: non-human cells

Many authors have used non-human cells as in vitro models for exploring radioprotective effects. Denissova et al. [37], for example, used mouse embryonic stem cells (mESC) to study the radioprotective effects of resveratrol. mESCs pretreated with resveratrol prior to irradiation had two-fold improved post-radiation viability compared to controls [37]. The resveratrol group was also shown to resume cell cycle progression sooner than irradiated controls [37]. Specifically, within 7.5-h post-radiation resveratrol pretreated cells had resumed transit through M phase to G1; however, the control cells did not [37]. Denissova et al. [37] postulated that resveratrol attenuated the G2 checkpoint; however, it did so without any observed increase in mutation frequency. This interesting finding suggests that resveratrol promotes earlier resumption of cell cycle transit after irradiation without compromising genomic integrity [37]. Remarkably, not only did resveratrol restore normal cell cycle function earlier, but DNA strand breakage repair was detectable sooner in irradiated cells pretreated with resveratrol [33]. In this study, resveratrol showed a noteworthy ability to improve the survival of irradiated cells without changing mutation frequencies or hampering the process of repairing DNA damage. Resveratrol is an agent with properties that should be investigated in other studies and settings.

Jelveh et al. [38] studied the effects of curcumin on skin fibroblasts in mice. They specifically tested eukarion, curcumin, and the curcumin analogs D12 and D68 on radiation-induced DNA damage in murine skin. Eukarion, curcumin, and the curcumin analogs did not show significant decreases in DNA damage when administered after radiation exposure; however, there was some decrease in DNA damage when they were given before irradiation [38]. While the effects of these agents on DNA damage were only observed with pretreatment, all the antioxidants did show significant protective effects against radiation-induced lipid peroxidation, even when administered after irradiation [38]. This study showed that, among these agents, only eukarion or curcumin had DNA protective effects and these effects were both minor and reliant on administration before radiation exposure [38]. Curcumin and its derivatives, then, may offer only minor DNA protection; however, it seems to offer significant protection against lipid peroxidation. More work should be done to further elucidate the mechanism by which curcumin derivatives are able to protect against lipid peroxidation after exposure to radiation.

Melatonin, a known radioprotective agent, was studied by Das et al. [39] in the setting of DNA plasmids. They found that DNA plasmids pretreated with melatonin showed a reduction in SSBs and DSBs in response to radiation [39]. The observed activity of melatonin in this study was dose-dependent, with 30% reductions of DSBs in low-dose pretreated cells and “virtually no detectable DSBs” in high-dose pretreated cells [39]. The efficacy of melatonin in these plasmids was preserved through exposure to various levels of radiation: more melatonin meant fewer SSB and DSB in pretreated plasmid DNA compared to controls [39]. Melatonin also reduced DNA damage from charged particles: plasmids in solution with melatonin treated with iron ions showed a 41% reduction in DSBs compared to controls [39]. They proposed that melatonin upregulates antioxidant enzymes, increases distribution of cells in S-phase and decreases G0/G1 cells, reduces lipid peroxidation, all of which could explain its protective mechanism [39].

### Findings from in vivo studies

#### In vivo: mouse model

Translational studies have also investigated NAC’s antioxidant abilities. One study found that pretreating mice with NAC decreased ROS by 4.8 times, improved 10- and 30-day survival, and maintained duodenal anatomy (relative to controls) [40]. Beyond quantifying the effect of pretreatment with NAC, this study also found that treating mice with NAC post-radiation provided a survival benefit compared to controls [40]. Other recent studies have also used murine models to study the benefits of NAC, and showed similar decreases in DNA damage [13].

Resveratrol has been investigated as a potential radioprotectant due to its antioxidant properties and its ability to scavenge free radicals and upregulate the activity of glutathione, superoxide dismutase, and catalase [31]. Additionally, resveratrol has been shown to induce apoptosis in cancer cells by caspase activation, upregulate p53 expression, decrease expression of anti-apoptotic proteins including BCl2 and induce expression of proapoptotic proteins including BAX expression [31]. Resveratrol also modulates cell cycle distribution, causing suppression of cell cycle progress and arrest of the cell cycle at key junction point [31]. Carsten et al. [31] performed a randomized controlled trial in which mice were given resveratrol and then irradiated to study its possible radioprotective effects in an animal model. Mice were given resveratrol prior to irradiation, and then daily in their drinking water for 30 days following irradiation with a 3 Gy whole body dose of gamma rays [31]. They found that mice treated with resveratrol before radiation exposure had a significant decrease in the total number of chromosome aberrations (fragments, gaps, dicentrics and robertsonian translocations) at 30 days’ post-radiation compared to irradiated controls [31]. In fact, the number of chromosomal aberrations in the resveratrol group was like mice who were not irradiated at all. These results suggest that resveratrol is effective at reducing total chromosome aberrations to background radiation levels 30 days after high dose radiation [31]. Possible explanatory mechanisms include resveratrol’s direct antioxidant properties, its indirect antioxidant effects through augmentation of glutathione, or resveratrol-mediated increases in expression of superoxide dismutase and catalase [31]. Resveratrol has also been shown to induce cell cycle arrest in S phase and/or G2/M transition in leukemia cells, potentially allowing more time for chromosomal repair following DNA damage [31].

Zhang et al. [41] investigated the effects of resveratrol against radiation-induced small intestine injury in mice. In this study, the mice were pretreated with resveratrol 5 days before and 1 day after prior to a large single dose partial-body (abdominal) irradiation. Mice were then sacrificed 6 days after irradiation and small intestines were examined for morphologic changes and crypt cell apoptosis. Pretreatment with resveratrol improved intestinal morphology, decreased crypt cell apoptosis, and improved expression of Ki-67, a marker for crypt cell [41]. The authors conclude that since intestinal tissue is particularly sensitive to ionizing radiation, resveratrol could be used to reduce damage to healthy small intestine tissues [41].

Pei et al. [10] investigated the effects of GANRA-5 in mice. Mice were given intragastric GANRA-5 for 25 days before irradiation with 8 Gy of X-rays. At 18 days’ post exposure the survival rate in the control group was 30%, while the GANRA-5 group was 85%. By 25 days GANRA-5 had a survival rate of 60%, with only 10% among irradiated controls [10]. The authors concluded that GANRA-5 likely acts as a free radical scavenger, thus reducing DNA damage and cell death after radiation exposure [10]. Additionally, GANRA-5 also reduced post radiation inflammatory response in mice, suggesting GANRA-5 may activate anti-inflammatory factors such as cyclooxygenase-2 [10].

Mohammad et al. [42] conducted an experiment in a mouse model that used low dose ionizing radiation to test the radioprotective activity of watermelon juice. They divided mice into two groups, one given tap water while the other was given 50% tap water plus 50% watermelon juice for 28 days. Following this period, both groups were exposed to total body irradiation. They found that watermelon juice diet supplementation significantly reduced lipid peroxidation in liver and lung tissue, but not brain tissues [42]. The authors also found that watermelon juice treated mice had significant reduction in DNA damage in brain, lung, and liver tissue compared to controls [42]. Watermelon juice treated mice also showed a significant increase in superoxide dismutase activity in the tissues of the lung, brain, and liver [42]. Finally, the watermelon juice group had increased glutathione levels in brain and liver tissues, but not lung tissue [42]. The authors concluded that the reduction in damage seen in this experiment was likely due to the high antioxidant content of watermelon juice, which is confirmed by measured increases in relative superoxide dismutase and glutathione in the treatment group [42].

Nishimura et al. [43] looked at the effects of lactoferrin as a radioprotective agent. They proposed that, since lactoferrin acts to chelate iron ions and inhibit hyperoxidation of lipids, it may provide broad protection against ionizing radiation. To test this hypothesis, they fed mice a diet high in lactoferrin while feeding the control group a lactoferrin-free diet. After 1 month, they exposed the mice to a single whole body dose of X-ray radiation. The lactoferrin mice had a higher 30-day survival rate (84.6%) compared to the control group (61.5%) [43]. They also found the lactoferrin group maintained higher body weights after 30 days compared to controls [43]. They then conducted an additional study where a group of mice were exposed to high dose of X-ray radiation and then injected with 4 mg of lactoferrin or saline. The lactoferrin treated group had a survival rate of 92% after 30 days, which was significantly higher than the saline treated group, which was 50% after 30 days [43]. The authors proposed that lactoferrin may offer radioprotective effects by hydroxyl radical scavenging activity, and by inhibiting hyperoxidation of lipids [43]. Like many other studies, this study highlights the importance of diet as a means of obtaining radioprotective antioxidants.

Recent studies have suggested that guanine nucleotides may be a preferred target for ROS on DNA and RNA [39]. Prior studies have also found that hydrolyzed RNA, specifically guanosine nucleotides, may act as a radioprotectant [44]. To test the radioprotective capacity of guanosine nucleotides, Asadullina et al. [44] either injected mice with 5 mM of GMP before radiation, injected mice with 5 nM of GMP after radiation, or provided no injection. They found that mice treated with GMP after irradiation had a nearly 40% improved survival at 30 days compared to both controls and those given GMP prior to irradiation [44]. They also found better leukocyte count recovery in mice given GMP after irradiation at 30 days compared to controls and the group given GMP prior to irradiation [44]. Platelet counts and granulocyte counts also remained higher and recovered faster in the GMP after radiation group compared to controls and GMP pretreatment group [44]. They also measured a reduction in hydroxyl radicals and hydrogen peroxide, suggesting an antioxidant effect or an upregulation in superoxide dismutase and/or glutathione [44]. Taken together, these results suggest that GMP may be particularly well suited as a mitigator of DNA damage, as the highest reduction in damage was seen when administered after radiation exposure.

Naeimi et al. [45] investigated potential effect of atorvastatin in irradiated mice as a potential radioprotectant in pelvic malignancy to reduce radiation damage to testicular tissue. In their experiment mice were given varying doses of atorvastatin 7 days prior to irradiation. Biochemical, histological, and immunohistological parameters were used to evaluate the potential radioprotective effects. Atorvastatin induced a dose-dependent protective effect, with the highest doses offering the most protection [45]. Mice pretreated with atorvastatin had a significant reduction in lipid peroxidation and higher concentrations of total serum testosterone [58]. Histologic examination showed a decrease in testicular epithelium thickness and atrophy of the seminiferous tubules in irradiated controls [45]. Mice pretreated with atorvastatin had increased epithelial thickness and seminiferous tubule diameter, however the authors point out the increased diameter of the seminiferous tubules was not statistically significant (p > 0.05) [45]. Mice pretreated with atorvastatin also showed decreased levels of caspase-3, suggesting atorvastatin may work by reducing apoptosis following irradiation [45].

#### In vivo: rat model

Prior research has suggested that polyphenols found in natural foods (such a grapes) have potential to act as a radioprotectant. While prior studies have focused on food extracts, Andrade et al. [46] wanted to know if whole food supplementation provide radioprotective effects in rats using black grape juice. Rats were fed ad libitum grapes and drank black grape juice or placebo 6 days before and 15 days following 6 Gy X-ray irradiation. Rats fed black grape juice showed a decrease in lipid peroxidation, increase in liver superoxide dismutase, and increase in glutathione peroxidase activity [46]. They also found that black grape juice supplementation resulted in reduced glutathione levels like those in non-irradiated rats [46]. They concluded that ad libitum black grape juice intake before and after X-ray radiation decreases liver lipid peroxidation, increases superoxide dismutase activity, and increases glutathione levels like that of food extract supplementation [46]. These results suggest that diet supplementation with foods high in antioxidants may play a role in reducing radiation induced DNA damage.

In another study investigating the radioprotective effects of phenolic glycosides, Chu et al. [47] conducted a study to evaluate the effects of caffeic acid phenethyl ester (CAPE) on upper abdominal radiation exposure in rats. They exposed rats to 30 Gy radiation to upper abdomen after being treated with CAPE. They found rats treated with CAPE had significantly less histologic alterations, lower ALT and AST levels, suggesting CAPE could protect against radiation-induced liver damage [47]. They found that CAPE pretreatment increased activity of superoxide dismutase and glutathione, suggesting that CAPE’s protective effects were due to (at least in part) balancing of pro-oxidant and antioxidant reactions in hepatic tissue [47]. Irradiated controls also had an increase in NF-kB p65 nuclear transport (a central mediator of immune response), however CAPE pretreated rats inhibited NF-kB’s ability to act as a transcription factor, thus reducing cascade of inflammatory response due to radiation [47]. As expected, there were also decreased levels of TNF-α and ICAM-1 expression through depression of NF-kB activation [47]. Finally, there was an overall decrease in apoptosis of hepatocytes pretreated with CAPE, suggesting it may have anti-apoptotic properties [47].

In one study, Zhang et al. [48] showed that irradiated rats pretreated with kukoamine A before irradiation demonstrated dose-dependent decreases in apoptosis. More specifically, these rats showed dose-dependent increases in anti-apoptotic mediators (like BCL2) and decreases in pro-apoptotic mediators (like BAX and caspase-3) as well as increased concentrations of antioxidants like superoxide dismutase and catalase [48].

Ortiz et al. [49] conducted a study to assess the effect of melatonin on irradiated rat oral mucosa. Melatonin gels of differing concentrations were then applied topically to the oral mucosa prior to radiation exposure. 3% melatonin gel ultimately showed the best results in decreasing mucositis [49]. Assays and studies on the tongues of irradiated rats showed that mitochondrial ROS production plays a role in mucositis and is influenced by the NF-kappa-B and NLRP3 activation (both are known to activate inflammatory pathways that increase the expression of genes responsible for causing mucositis) [49]. The authors concluded that is a likely connection between mitochondrial impairment and activation of the innate immune system, which may contribute to the development of mucositis [49]. Since melatonin acts as an anti-inflammatory by modifying expression of NFkB and NLRP3 it may be a promising candidate as a radioprotective agent against oral mucositis [49]. Other research has suggested that melatonin may also increase the expression of antioxidant enzymes such as superoxide dismutase and glutathione peroxidase [50, 51].

Sridharan et al. [52] conducted a study looking at tocotrienol, a vitamin E analog and its ability to reduce radiation induced cardiac damage in rats. In their experiment, they gave rats high dose tocotrienol via oral gavage or a control 24 h before local heart irradiation. After pre-treatment they irradiated the rats with high dose localized radiation to their hearts. They found that rats pretreated with tocotrienol did not have significantly elevated Bax/BCL2 ratios compared to non-irradiated controls [52]. They also found that the mitochondria of rats pretreated with tocotrienol maintained the mitochondrial membrane potential without increase swelling commonly seen in cell death [52]. Tocotrienol treated groups also showed significantly reduced cleaved caspase 3 levels at 2 and 28 weeks [52]. To better understand the mechanism, the authors measured levels of reduced glutathione (GSH) and oxidized glutathione (GSSG) and calculated a GHS/GSSG ratios (with a decreased ratio indicating significant oxidative stress) [52]. They found that rats pretreated with tocotrienol showed GSH/GSSG ratios that did not differ significantly from non-irradiated controls, suggesting overall reduction in oxidative stress which may lead to the protective effects against mitochondrial damage [52]. They concluded that tocotrienol before radiation was effective at maintaining proapoptotic Bax levels and anti-apoptotic Bcl2, likely due to reduced oxidative stress [52].

Vasil’eva et al. [53] conducted a study to assess the radioprotective effects of alpha-tocopherol acetate (TA), ascorbic acid (AA), or a combination of these agents in rats. They found that a combination of TA and AA administered before and after radiation protected the bone marrow from radiation induced changes [53]. Although a combination of TA and AA reduced damage, either agent administered separately before or after irradiation did not affect the frequency of chromosome aberrations compared to controls, suggesting that the agents acting alone do not offer radioprotective effects [53]. Combinations of TA and AA given together 1 h or 10 min before, and 10 min or 3 h after significantly reduced the frequency of chromosome aberrations by 2–2.5 times in comparison with controls [53]. Given that combinations of TA and AA showed a radioprotective effect, authors hypothesized that the two antioxidants may have a synergistic or antioxidant regenerative effects [53].

Because previous studies have suggested that sex hormones such as estrogen have shown to have neuroprotective properties in animal models with focal and global cerebral ischemia, Caceres et al. [54] conducted an experiment to evaluate what effects of 17β-estradiol has on the hippocampus of neonatal rats exposed to ionizing radiation. In their experiment, they randomized rats into two categories, estrogen treated and placebo treated. They further divided the estrogen treatment group into treated before and after radiation exposure. The rats were exposed to high dose X-rays between 24 and 48 h after birth. Hippocampal ROS levels and protein kinase C activity were assessed, as ROS are known activators of PKC [54]. They found that rats given estrogen before irradiation had normal levels of hippocampal ROS when compared to controls, suggesting estrogen may influence mitigation of free radical formation and propagation [54]. The authors proposed that 17β-estradiol may act via an antioxidant mechanism, thus reducing the propagation of reactive oxygen species. They further proposed that high estrogen levels could act as a direct free radical scavenger [54]. Thus, it was inferred that 17β-estradiol may reduce DNA damage caused by ionizing radiation by reducing reactive oxygen species propitiation and reducing the available free radicals that could interact with DNA [46]. Although 17β-estradiol could reduce ROS, administration failed to prevent increase in protein kinase C activity [54]. The authors proposed that this may be due to differential regulation of PCK isoforms [54]. Preliminary data has shown that PKC-B1 levels are upregulated by ROS. The authors suggest that 17β-estradiol may still upregulate PKC isozymes differently, that is increasing some while decreasing others resulting in little overall change to PKC activity [54], however future studies are needed to confirm this assertion [54].

Huang et al. [55] conducted an experiment using amifostine (a radioprotector currently available in clinical practice) 30 min prior to a lethal whole abdominal dose radiation in Sprague–Dawley rats. Some rats were sacrificed to determine p53 expression and crypt cell survival, and others were observed to determine changes in survival based on amifostine administration. Rats given amifostine had improved survival rate, with an overall survival rate of 90% (compared to 0% in control groups) [55]. Interestingly rats given p53 inhibitors and amifostine did not have improved survival compared to controls, suggesting amifostine acts (at least in part) via a p53 dependent mechanism [55]. The authors found that amifostine administration significantly increase p53 accumulation in the nucleus [55]. Additionally, rats given amifostine had decreased mucosal damage, improved regeneration, and improved crypt cell survival compared to controls [55]. Like survival rates, rats given a p53 inhibitor and amifostine did not have improved mucosal survival, regeneration, and crypt cell survival [55]. The authors propose that amifostine may enhance p53-dependent protective effects by increasing nuclear accumulation, inhibiting degradation, and inducing transcription factors related to p53 expression [55].

#### In vivo: human

In a 2017 publication Velauthapillai et al. [56] conducted a prospective controlled trial to assess the effectiveness of a multi-agent oral antioxidant pill as a radioprotectant. This pill contained ascorbate, NAC, lipoic acid, and beta carotene [56]. The study patients received the pill before clinically-indicated Tc99 m scans for cancer staging. The number of DNA DSBs was assessed both before and after the imaging by looking at gH2AX foci in blood mononuclear cells. The baseline level of DNA damage was similar between treatment and control groups before the bone scan [56]. While the sample was not large, (five in the treatment group and five in the control group) this study found a significant reduction in DSBs in the treatment group compared to controls [56]. The antioxidant treatment group did not have a significant difference in the total number of DSBs before and after imaging [56]. Meanwhile, the median number of gH2AX foci per cell rose significantly in the control group [56]. The authors found that treatment with antioxidants accounted for nearly 60% of the difference between in DNA damage treatment and control groups after the scan [56]. Furthermore, NAC and ascorbate peak in blood concentrations 2.5 h after ingestion, which is when the cells were drawn from patients for evaluation, suggesting that NAC and ascorbate may be playing a larger role in radioprotection [56]. Although this study showed reduced ionizing radiation damage in humans after a nuclear medicine scan, the sample size was small, and more research will need to be done using different imaging modalities. Further studies investigating the effects of radioprotective agents on human subjects, particularly those that explore long-term effects, are certainly necessary.

One interesting trial was conducted using high dose melatonin as a radioprotector in a phase II radiation therapy oncology group trial in patients with brain metastases [51]. In this trial, the patients were randomized into two categories, and administered with 20 mg of melatonin or a placebo in the morning or evening. All patients received whole brain radiation treatment in the afternoon. Neither group had statistically significant survival, and it was concluded that oral melatonin did not show any beneficial effect in this study [51].

Although the literature supports many potential radioprotective agents for clinical use, there are currently few agents approved for clinical use in the United States. Two well-known examples are amifostine and palifermin [57]. Amifostine is sulfhydryl compound and a free radical scavenger and is currently used as a radioprotectant during radiotherapy, while palifermin acts to suppress apoptosis and is used to reduce mucositis [57]. Amifostine has also been shown to accumulate more rapidly in normal tissues than malignant cells, making it an ideal radioprotectant for radiotherapy [50]. Randomized trials of amifostine as a radioprotector showed a reduction in late xerostomia, mucocitis, dysphagia, dermatitis, pneumonitis, proctitis, and cystitis [50]. Like other sulfhydryl compounds, amifostine is thought to work primarily via free radical scavenging and up regulation of existing enzymes superoxide dismutase and glutathione peroxidase [50], however, some authors have proposed it may work via a p53-dependent mechanism [55].

## Discussion

Literature exploring a potential role of radioprotective agents is growing rapidly. There are three important caveats related to the body of research on radioprotective agents: study radiation doses, uncontrolled exposure to radioprotective agents (via diet), and long term implications. First, many researchers subject test animal and cellular models to much higher doses of radiation than those used during medical imaging. This tendency may enable studies to identify exaggerated radioprotective effects. Despite these differences, research has shown that even low doses of ionizing radiation produce cellular responses (double stranded breaks, free radical formation, lipid peroxidation, cellular necrosis, apoptosis) that are analogous, albeit smaller, than larger radiation doses [58]. In a 2011 study authors measured DSB in human lymphocytes after a CT angiography and found a significant increase in DSBs 30 min after the scan was complete [58]. For this reason, radioprotectants may still be beneficial. Second, many of the radioprotectant agents studied in the literature are available widely available in diets, particularly healthy diets. The possible presence of these agents in potential study populations could confound research on the effectiveness of radioprotectant agents. Third, while radiation exposure has been linked to DSBs and other types of DNA damage, there is no long-term data that proves a reduction in DSBs leads to a reduction in carcinogenesis and teratogenesis. In other words, there may be little actual clinical value in preventing imaging-associated DSBs with radioprotective agents.

Regardless of these caveats, there seems to be sufficient evidence to warrant investigation of a potential clinical role for radioprotective agents. While radioprotectant agents have not yet been definitively proven to have measurable clinical benefit, radiation exposure is known to have undesirable clinical sequelae. Significantly, a 2009 study estimated that 2% of cancers (and an associated 15,000 deaths annually) can be linked to CT exposure [59]. While it remains to be seen whether radioprotective agents could prevent these cancers and deaths, the existing literature does support the capacity of radioprotective agents to decrease radiation-associated damage on a molecular level. Moreover, radioprotective agents, which are largely available in a healthy diet, pose little risk to patients in most cases. In other words, these agents may potentially prevent radiation-associated complications while posing little (if any) intolerability or negative effects.

Many authors proposed that the respective radioprotective agents work by increasing natural antioxidants’ abilities, or by acting as direct free radical scavengers (Fig. 1) [50]. Some agents have proposed cell cycle and apoptosis pathway effects (Fig. 3), although it is possible that others may affect the cell cycle as well. Given well-studied effects of ionizing radiation on human cells [1], and the increasing utilization of imaging in modern medicine, a method to reduce cell damage caused by ionizing radiation has the potential to be beneficial in reducing morbidity and mortality. The literature contains sufficient evidence to suggest that pre- and/or post-treatment of patients with radioprotective agents may decrease damage due to ionizing radiation. Regardless of these observed effects, more research is needed to determine whether radio-protective interventions would offer long-term benefit or decreases in toxicity in clinically relevant populations.

Inflammatory injury is another field in which radioprotective agents show promise. Interestingly, inflammatory injury is associated with exhaustion of cellular antioxidants. Some studies suggest that radioprotectants reduce cellular damage (and possibly even cell death) by reducing the post radiation inflammatory response [18, 23, 47, 49]. Although the mechanism by which oxidizing agents is distinct from ionizing radiation, conditions like severe sepsis and septic shock also create an oxidation-rich environment that leads to the depletion of natural cellular antioxidants (such as glutathione) and contributes to cellular injury and death. The oxidant-rich environment created by conditions like severe sepsis and septic shock, then, is analogous in some ways to the conditions that arise in response to ionizing radiation. It has been suggested that inflammatory injury may be the result of inadequate antioxidant protection [51]. Moreover, at least one study has shown that antioxidants may play a role in protecting patients from inflammation-related cell damage in the ICU [60]. Thus, radioprotectants may have greater application in reducing inflammatory injury beyond what occurs in response to radiation exposure. More research is needed to determine future clinical applications in this area.

Some studies have shown that dietary supplementation with foods high in antioxidants can reduce DNA damage, lipid peroxidation, and improve survival following high dose radiation exposure [17, 29, 42, 43, 46]. Authors of these studies have suggested that supplementation with food high in antioxidants may be a means of radioprotection. In other words, proper nutrition may be the only necessary defense against imaging-related radiation exposure.

## Conclusion and future directions

Radioprotective agents reduce DNA damage, as evidenced by findings under in vitro, in vivo, and in human randomized controlled trials. Their use in clinical medicine to reduce DNA damage and lipid peroxidation may lead to a reduction in carcinogenesis and teratogenesis and may improve patient morbidity and mortality. Although radioprotective agents theoretically should reduce carcinogenesis and teratogenesis, we could not find long-term trials that show that radioprotective agents prevent long-term stochastic effects of radiation exposure (like cancer). One area of interest is that radioprotective agents are often phytonutrients that are found in a well-balanced diet, particularly in plant-based diets. This observation suggests that diet modification alone could provide radioprotective effects. Many of the agents that have been investigated and found to have radioprotective potential are both inexpensive and well-tolerated. Although many of the radioprotectants discussed in this article may up-regulate natural antioxidants or as direct free radical scavengers, this is not the only mechanism by which ionizing radiation produces DNA and cellular damage. Further research is warranted to determine exact mechanisms of radioprotection, which may help us better identify radioprotective agents. While long-term research should be done to establish the clinical value of radioprotective agent use in the setting of medical imaging, the harm and cost of adding these agents is negligible.

Based on the critical evaluation of the findings in the literature, we hypothesize that providing appropriate doses of radioprotective agents before medical imaging would pose little harm to patients and would carry the potential for significant clinical benefit.

### Key points

- Currently radioprotective agents are not used routinely in diagnostic imaging.
- Many diagnostic imaging modalities utilize ionizing radiation to generate clinical information. Ionizing radiation can cause direct damage to proteins, DNA, and lipid membranes. Further, ionizing radiation can induce free radical formation and indirectly damage DNA, proteins, and lipids through this mechanism.
- Radioprotective agents have shown to reduce DNA damage in vitro, in vivo, and in human randomized controlled trials.
- Cellular and animal model research suggests that radioprotective agents can reduce DNA damage through various mechanisms, however most of the available information in the literature suggest that free radical scavenging and induction of natural antioxidants likely play a role.
- Radioprotective agents have been shown in histologic studies to decrease cell damage and increase post radiation cell proliferation.
- One study showed a decrease in double stranded breaks in human lymphocytes in patients who ingested an radioprotective combination before a diagnostic imaging scan.
- Some agents have been shown to have direct cell cycle effects as a proposed mechanism of radioprotection. It is likely that other agents do as well, but this area needs more research.
- Radioprotective agents may lead to a reduction in carcinogenesis and teratogenesis through their effects on DNA damage, lipid peroxidation, protein damage, and cell cycle regulation.
- Although radioprotective agents theoretically should reduce carcinogenesis and teratogenesis, we could not find long-term trials that show that radioprotective agents prevent long-term stochastic effects of radiation exposure (like cancer).
- One area of interest is that radioprotective agents are often phytonutrients that are found in a well-balanced diet, particularly in plant-based diets. This observation suggests that diet modification alone could provide radioprotective effects.
- While long-term research should be done to establish the clinical value of radioprotective agent use in the setting of medical imaging, the harm and cost of adding these agents is negligible. Based on the findings of this review, we hypothesize that providing appropriate doses of radioprotective agents before medical imaging would pose little harm to patients and would carry the potential for significant clinical benefit.

## Abbreviations

CT: computed tomography
ROS: reactive oxygen species
SSBs: single stranded breaks
DSBs: double stranded breaks
γH2AX: gamma-H2AX foci
NAC: *N*-acetylcysteine
PlmtVC: 6-*O*-palmitoylascorbate
l-AA: l-ascorbic acid
CVC: carvacrol
sEg: sinapoyl-E-glucoside
q3Or7Og: quercetin-3-*O*-rhamnoside-7-O-glucoside
q3Or: quercetin-3-*O*-rhamnoside
l7O2ag: luteolin-7-*O*-(2-apiosyl)-glucoside
DNC: dendrosomal nanoformulation of curcumin
DA: dendrodoine analog
EGCg: epigallocatechin-3-gallate
HO1: oxygenase 1
IF: isofraxidin
mESC: mouse embryonic stem cells
CAPE: caffeic acid phenethyl ester
GHS: glutathione
GSSG: oxidized glutathione
TA: α-tocopherol acetate
AA: ascorbic acid
